# Process evaluations for cluster-randomised trials of complex interventions: a proposed framework for design and reporting

**DOI:** 10.1186/1745-6215-14-15

**Published:** 2013-01-12

**Authors:** Aileen Grant, Shaun Treweek, Tobias Dreischulte, Robbie Foy, Bruce Guthrie

**Affiliations:** 1 Quality, Safety and Informatics Research Group, Population Health Sciences, Medical Research Institute, University of Dundee, Mackenzie Building, DD2 4BF, Dundee, UK; 2 Medicines Management Unit, NHS Tayside, c/o University of Dundee, Mackenzie Building, DD2 4BF, Dundee, UK; 3 Leeds Institute of Health Sciences, Charles Thackrah Building, University of Leeds, 101 Clarendon Road, LS2 9LJ, Leeds, UK

**Keywords:** Process evaluation, Complex intervention, Cluster-randomised controlled trial, Qualitative, Quantitative, Reporting

## Abstract

**Background:**

Process evaluations are recommended to open the ‘black box’ of complex interventions evaluated in trials, but there is limited guidance to help researchers design process evaluations. Much current literature on process evaluations of complex interventions focuses on qualitative methods, with less attention paid to quantitative methods. This discrepancy led us to develop our own framework for designing process evaluations of cluster-randomised controlled trials.

**Methods:**

We reviewed recent theoretical and methodological literature and selected published process evaluations; these publications identified a need for structure to help design process evaluations. We drew upon this literature to develop a framework through iterative exchanges, and tested this against published evaluations.

**Results:**

The developed framework presents a range of candidate approaches to understanding trial delivery, intervention implementation and the responses of targeted participants. We believe this framework will be useful to others designing process evaluations of complex intervention trials. We also propose key information that process evaluations could report to facilitate their identification and enhance their usefulness.

**Conclusion:**

There is no single best way to design and carry out a process evaluation. Researchers will be faced with choices about what questions to focus on and which methods to use. The most appropriate design depends on the purpose of the process evaluation; the framework aims to help researchers make explicit their choices of research questions and methods.

**Trial registration:**

Clinicaltrials.gov NCT01425502

## Background

Many interventions to improve healthcare delivery and health are complex in the sense that they possess several interacting components [1]. Randomised controlled trials of such interventions are often criticised as being ‘black box’, since it can be difficult to know why the intervention worked (or not) without examining underlying processes. As a result, reported effects of what appear to be similar interventions often vary across studies because of differences in context, targeted groups and the intervention actually delivered [2,3]. The UK Medical Research Council guidance for developing and evaluating complex interventions recommends conducting a process evaluation to ‘explain discrepancies between expected and observed outcomes, to understand how context influences outcomes, and to provide insights to aid implementation’ [1]. Although our focus is on randomised trials, it is important to recognise that similar issues arise in the evaluation of public health interventions which may use a range of different evaluative designs [4-6].

In complex intervention trials, the intervention to be evaluated may be delivered at different levels: the intervention can be delivered straight to the patient, such as in trials evaluating the effectiveness of body weight and physical activity interventions on adults [7]; the intervention can be delivered to the healthcare professional, for example in trials of decision support tools [8]; or the intervention can be targeted at the organisational level, such as in a recent trial testing a pharmacist-led information-technology-based intervention aimed at optimising medication safety systems in general practice [9] (adapted from the Medical Research Council guidance 2000) [10].

Process evaluations are studies that run parallel to or follow intervention trials to understand the trial processes or underlying mechanisms in relation to context, setting [11], professionals and patients [12]. These evaluations provide explanations for the trial results and enhance understanding on whether or how interventions could move from research to practice.

We planned to conduct a parallel process evaluation of a cluster-randomised trial of a complex intervention to improve prescribing safety [13] targeted at general practices. Although there is clear published guidance for designing trials where clusters rather than individuals are the unit of randomisation [14,15], we found the Medical Research Council guidance of limited help in designing the process evaluation of our trial.

In our trial, the research team delivers an intervention to general practices – with the intention of prompting those practices to review patients with particular types of high-risk prescribing (nonsteroidal anti-inflammatory drugs or antiplatelets), to carefully reconsider the risks and benefits of the offending drugs in each patient and to take corrective action where possible [13]. There is therefore an intervention delivered to practices (the cluster) that hopes to change the behaviour of practitioners. This approach is similar to most trials of audit and feedback [16] and to many trials involving a professional training intervention, as in the OPERA trial where a training intervention is delivered to nursing home staff and physiotherapists who deliver an intervention of physical activity and physiotherapy to residents of residential and nursing homes [17,18]. In other trials, however, the intervention is delivered to individuals by the research team themselves, as in a trial of body weight and physical activity for adults at risk of colorectal adenomas [7]. Process evaluations therefore need to be tailored to the trial, the intervention and the outcomes being studied, but we could not find clear guidance in the literature on how to do this. Additionally, we noted wide variations in the aims, methods, timing and reporting of process evaluation studies that made it difficult to draw on them in designing our own process evaluation.

We therefore developed a framework to guide the design of our own process evaluation. We believe it may be of help to other cluster triallists faced with a similar task. We also make proposals for the reporting of process evaluations to enhance their identification and usefulness to other researchers.

## Methods

### Existing literature

To support the design of our own process evaluation, we wanted to identify relevant methodological and theoretical literature. We did not aim for a comprehensive review of all such studies but rather sought to gain a general understanding of this literature. We were particularly interested in the purpose and the design of process evaluations of cluster-randomised controlled trials. Our initial search (which included the terms ‘process evaluation’ or ‘qualitative’ or ‘quantitative’ and ‘complex intervention’ or ‘randomised controlled trial’) in Medline, Embase, Psycinfo, Cochrane Central Register of Control Trials and Cochrane Database of Systematic Reviews was informative but identified studies that tended to focus on a specific aspect of process evaluation design or the nature of process evaluations and qualitative methods. There was little guidance on how to design a process evaluation. We turned to process evaluations from cluster-randomised controlled trials identified by our search from which we could learn. We designed a data extraction form based on the Reach, Efficacy, Adoption, Implementation and Maintenance (RE-AIM) framework [19] and Northstar [20] to summarise identified reports of process evaluations in terms of: the aim of the work; the timing (parallel to the trial or *post hoc*); its scope with reference to each RE-AIM construct (reach, efficacy, adoption, implementation and maintenance) and the methods used; and trial and process evaluation findings and how they were reported. This form was systematically populated with information from all identified process evaluations by AG, with a second review of each by either ST, RF or BG. Disagreements between reviewers were collectively discussed and resolved, and general strengths and limitations in design and reporting were identified.

We initially set out to design a critical appraisal form but there was wide variation in the reporting of process evaluations that made consistent critical appraisal difficult. We struggled to make judgements based on the breadth of information reported in our data extraction form because we found studies that chose to report one process in detail but did say if other processes had been considered and an explicit judgement had been made not to evaluate them [21]. Some general features of this literature are summarised below.

## Results and discussion

### Process evaluation purpose

The generally agreed purpose of process evaluations of trials of complex and public health interventions is to understand the effects (or not) of interventions [2-4]. These effects can help to inform judgements about the validity by delineating key intervention components (construct validity), and by demonstrating connections between the intervention and outcomes (internal validity) and between the intervention and other contexts (external validity) [4,22]. Implementation and change processes can be explored [1,23], and factors associated with variations in effectiveness can be examined [24]. Process evaluations can also examine the utility of theories underpinning intervention design [25] and generate questions or hypotheses for future research. Process evaluations can therefore fulfil a variety of purposes, including understanding intervention effects, potential generalisability and optimisation in routine practice [26].

### Process evaluation design

Published papers on trial process evaluation design largely focus on qualitative research methods [3,23,27], but many of the purposes identified above can be assessed both quantitatively and qualitatively; indeed, process evaluation of public health interventions often explicitly uses mixed methods [4]. The focus on qualitative methods in trials may occur because many routinely collect some quantitative process data measures to fulfil Consolidated Standards of Reporting Trials (CONSORT) requirements or as secondary outcomes, rather than as part of a distinct process evaluation. For example, data on recruitment are likely to be collected and reported as part of the main trial because reporting these data is a CONSORT requirement [28], but for many trials a more detailed examination of the process of recruitment may be useful, to inform interpretation of trial results and generalisability.

### Process evaluation reporting

We found it difficult to systematically identify process evaluations of complex intervention trials, and the reporting of important information was also variable. Although some papers were easily identifiable and had clearly stated purposes and methods [21,29,30], others:

• were hard to identify because of a lack of explicit labelling: 

• lacked clarity about their overall purpose or did not state specific objectives: 

• did not clearly state whether the evaluation was pre-planned and carried out in parallel to the main trial or was conducted *post hoc*: 

• did not provide an explicit statement of the main trial findings, making it difficult to judge the relevance and implications of the process evaluation.

Overall, although the existing literature was often thought-provoking, it did not provide an over-arching framework for designing process evaluations.

### Framework for designing process evaluations of cluster-randomised trials of complex interventions

In developing our framework we drew upon the general literature on process evaluation and the RE-AIM framework [3,11,19,23,26,27,31], and on published process evaluations [11,12,21,29,32,33]. The framework was developed by all the authors through iterative meetings, redefining and working with the concepts and testing this work against published evaluations.

**Figure 1:**
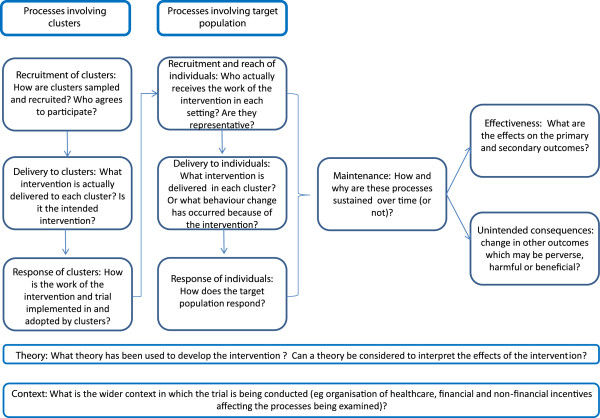
**Framework model for designing process evaluations of cluster**-**randomised controlled trials.**

Cluster-randomised designs are utilised when individual randomisation is not possible, feasible or appropriate, mainly because of potential contamination between the intervention and control arms [34]. Following the most recent CONSORT extension for cluster-randomised trials [28], we distinguish between individuals in the target population on whom outcome data are collected and ‘clusters’, which are some larger social unit within which individuals are nested and where the cluster is the unit of randomisation. Clusters may be defined by geography, institution, teams or services within institutions, or by individual professionals delivering a service to patients. Although outcomes are collected on an individual level, the primary unit of inference of the intervention may be the individual or the cluster, depending on the purpose of the study and the primary outcome measures. The focus of a process evaluation will vary with the nature of the trial and resources available, so although several key processes are candidates for examination in any evaluation of cluster trials (Figure 1), the importance of studying them will vary between trials. Also important to note is the fact that although the emphasis in what follows is on processes in clusters that receive the intervention, it will usually be equally important to understand relevant processes in control or usual care clusters.

### Recruitment of clusters (areas, institutions, individual professionals)

How clusters are sampled and recruited is important to help understand how generalisable the findings are likely to be. At a minimum, recruitment is likely to be monitored to fulfil CONSORT requirements for reporting findings of the main trial [28], but a more in-depth understanding of why clusters participate (or not) can inform the interpretation and implementation of trial findings.

### Delivery to clusters

In some trials, the research team or specially trained professionals may deliver a complex intervention to institutional or individual professional clusters, which are then expected to change their practice or service delivery in some way that impacts on the individuals in the target population. Understanding the nature of cluster-level interventions and variations in their delivery may help explain variation in outcomes between organisational clusters.

### Response of clusters

Response is the process by which clusters such as institutions or individual professionals integrate and adopt new trial-related or intervention-related work into their existing systems and everyday work, and will be influenced by how the research team engages and enrols the clusters and by the extent to which the work is perceived to be legitimate within organisations or by individual professionals [35].

### Recruitment and reach of individuals

Interpretation of the intervention’s measured effectiveness in the trial will depend on recruitment and reach, in terms of the proportion of the target population that actually receives the intervention, and how representative they are. Variation in the response of clusters will affect how clusters identify and enrol these individuals. When recruitment of individuals is carried out by organisational or individual professional clusters, there is potential for selection bias [34] – although similar issues arise in clusters defined by geography, where a variable proportion of the population may receive a public health intervention [4]. As well as influencing effectiveness, if high or representative recruitment and reach is not achieved within clusters in the trial, then the population impact of implementation in routine practice is likely to be limited.

### Delivery to individuals

In some studies, a complex intervention is delivered to the target population of individuals. Such interventions may be tightly defined by the researchers [36], in which case evaluation of delivery to individuals in the target population may focus on how closely the intervention delivered matches what the researchers intended (usually called the fidelity of the intervention) and the impact of deviation on effectiveness. In some trials, such as those on guideline implementation strategies, the intervention is not directly delivered to individuals (patients) but instead aims to change professional behaviour. The nature of the changes to work patterns or professional behaviour at the individual or patient level can be examined to better understand why it succeeded or failed. For example, in our trial the Data-driven Quality Improvement in Primary Care (DQIP) complex intervention is targeted at general practitioners to improve prescribing safety. This intervention prompts practitioners to carry out a targeted medication review based on a guideline provided to them, but practitioners are free to review medication in any way they judge appropriate [13]. Understanding how practitioners actually carry out these medication reviews is therefore likely to be important to explaining the trial results.

### Response of individuals

The effectiveness of many but not all interventions will be mediated by the responses of the targeted individuals, particularly where effectiveness is strongly influenced by adherence or requires behaviour change in those individuals. Similar to understanding the response of organisational or individual professionals to interventions directed at them, it may be helpful to draw on prior [37] or other relevant theory [38] to examine individual responses.

### Maintenance

Understanding whether and how each of the above processes is maintained or sustained over time is important, especially for studies of longer duration. Organisational clusters or individuals may cease to participate completely or the way in which they adopt and respond to the intervention may change over time, such as with declining reach or changes in the nature of the intervention delivered.

### Effectiveness

Although pre-specified measurement of effectiveness is the primary function of the main trial, the process evaluation can examine associations between trial processes and effects on primary and secondary outcomes – recognising that these are exploratory analyses. This can help explain why an intervention did or did not work, and can explore variations in effectiveness between clusters that may be important in planning more widespread implementation.

### Unintended consequences

All interventions have the potential to produce unintended consequences, which may be beneficial or harmful. Process evaluations provide an opportunity to systematically identify and quantify unexpected unintended outcomes.

### Theory

If theory has been used to develop the intervention, then this should be made explicit, and the process evaluation can examine whether predicted relationships and sequences of changes happen during implementation. Causal modelling based on psychological theory is an example that would apply to ‘delivery to the individual’ and ‘response of individuals’ [37]. Even if interventions do not have a strong theoretical base, then it may be useful to draw on relevant theory to help understand their effects [35].

### Context

Irrespective of which specific processes are examined, a careful description of the context in which the trial is embedded will usually be helpful for interpreting the findings, including how generalisable any findings are [39]. Additionally, trials are often conducted in different contexts – for example, within different health boards or different countries – and so consideration of these local contexts may be important. Each of the concepts in the model has contextual factors that may act as barriers and/or facilitators to implementation. For example, the DQIP trial is delivered in two health boards within Scotland that have different pre-existing levels of prescribing improvement support for practices, and this support has a different focus in each board. As well as the wider context of the organisation of primary healthcare in the United Kingdom being important, these local contextual factors may modify how practices respond to the same intervention [13,40].

### Applying the framework to different types of trial

As described above, cluster-randomised trials vary greatly in their design, including the level at which interventions are targeted. The framework can therefore be seen as a set of candidate elements for designers of process evaluations to consider. The relevance and relative importance of candidates will vary between trials, the aim and objectives of the evaluation and whether the authors plan to pre-specify their data collection and analysis (potentially allowing hypothesis testing) or conduct the evaluation *post hoc* (for example, depending on the experience of conducting the trial or on the trial results, in which case the evaluation can only be hypothesis generating). Inevitably, resource limitations will often require a focus on elements that are considered most critical in the context being evaluated. In such cases, emphasis should be placed on collecting quality data for a few key processes rather than collecting a lot of data for each candidate process. The following section discusses how the framework may be applied.

### Research methods for process evaluations of cluster-randomised trials

The appropriate methods for a process evaluation will depend on the intervention being evaluated and the aims of the evaluation. Table 1 presents examples of research questions that could be asked within each domain and methods to answer these questions. Both qualitative and quantitative methods can be appropriate depending on the questions asked. Quantitative data collection will be required if it is judged that all clusters should be evaluated in large multisite trials [3], whereas sampling may be necessary for more in-depth qualitative evaluation. Mixed methods can add complementary insights. For example, how clusters vary in their response and delivery of an intervention to the target population could be examined using qualitative analysis of interview and observational data, with variation in outcome across clusters examined quantitatively. Similarly, a study that seeks to explore individuals’ receipt of and response to an intervention can initially use quantitative data to inform sampling strategies and qualitative methods to explore different experiences (potentially at different stages of implementation). For some purposes, explicitly theoretical approaches to design and analysis will be appropriate, such as the use of psychological theory to understand individual behaviour [21], or sociological approaches to understanding response in different contexts, such as realistic evaluation or normalisation process theory [11,35]. Even without explicit use of theory, however, a process evaluation can productively draw on the researchers’ implicit models of how an intervention is expected to work [30,41]. Although the right design and method will therefore depend on the purpose of the evaluation, which will vary with the trial design and context, it is critical that the choices made and the rationale behind them is made explicit.

**Table 1:** Example research questions and methods for evaluating each process

**Domain**	**Example research questions**	**Research methods that could be applied**	**Probable best stage of study to collect data**
Recruitment of clusters	How are clusters sampled and recruited?	Documentation of recruitment process by research team.	Pre-intervention
	Who agrees to participate?	Quantitative comparison of recruited and nonrecruited clusters.	
	Why do clusters agree to participate (or not)	Qualitative analysis of interviews with cluster gatekeepers or members.	
Delivery to clusters	What intervention is actually delivered for each cluster? Is it the one intended by the researchers?	Qualitative analysis of observational, interview and documentary data relating to the cluster-level intervention.	Pre-intervention and early intervention
Response of clusters	How is the intervention adopted by clusters?	Quantitative data measuring cluster members’ perceptions of the intervention and uptake of trial components. Qualitative analysis of observational, interview and documentary data about how clusters adopt the intervention.	Pre-intervention and early intervention
Recruitment and reach in individuals	Who actually receives the intervention in each setting? Are they representative?	Measurement of receipt in target population. Quantitative comparison of those receiving vs. not receiving the intervention.	During intervention
	Why do clusters achieve the pattern of reach they do? Do they introduce selection bias?	Qualitative analysis of observational, interview and documentary data about how clusters achieve reach.	
Delivery to individuals	What intervention is actually delivered for each cluster?	Qualitative analysis of observational, interview and documentary data about what intervention is delivered and why.	During intervention
	Is the delivered intervention the one intended by the researchers?	Measurement of intervention fidelity across its components.	
Response of individuals	How does the target population respond?	Qualitative analysis of observational and interview data about target population’s experience of and response to the intervention.	During intervention and post-intervention
Maintenance	How and why are these processes sustained over time (or not)?	Any of the above, but probably focused on processes identified as critical, or as likely to be difficult to sustain.	During intervention and post-intervention
Unintended consequences	Are there unintended changes in processes and outcomes, both related to the trial intervention and unrelated care?	Qualitative analysis of observational and interview data for identification. Quantitative data collection for potential unintended consequences during the trial, or use of routine datasets.	Intervention and post-intervention
Theory	What theory has been used to develop the intervention?	Quantitative process data analysis can assess whether predicted relationships and sequences of change happened during implementation.	Post-intervention
Context	What is the wider context in which the trial is being conducted?	Qualitative data collection from policy documents or interviews.	Pre-intervention and early intervention

### Reporting process evaluations

The framework systematically identifies cluster-randomised trial processes that are candidates for evaluation. In practice, choices will often have to be made that balance the ideal with the feasible, focusing attention and available resources on key research questions. More systematic reporting of key information would help make the rationale for choices more explicit, improving interpretation. We believe that this key information includes the following factors:

*Process evaluations should be clearly labelled.* Existing process evaluation studies are poorly labelled and hard to identify.

*Process evaluations should clearly state their purpose.* Process evaluations should explicitly state their original purpose and research questions, the processes being studied and an acknowledgement of what is not being evaluated [21]. Changes to research questions during the study can be legitimate but should be explicit. For example, a pre-planned examination of recruitment and reach may prove to be less important than understanding unexpected variation in intervention delivery.

*Process evaluations should clearly report if they were pre-specified or post hoc, and why the selected timing was chosen.* Pre-specified and *post-hoc* process evaluations are both legitimate designs. Pre-specified evaluations are more suited to quantitatively examine prior hypotheses about trial processes, whereas *post-hoc* evaluations are more flexible for examining unanticipated problems in implementation or unexpected findings. Again, clarity in what was done and why is key to interpreting the validity and credibility of the findings.

*Process evaluations should state the choice of methods and justify them in terms of the stated aims of the evaluation*. The rationale for the methods used should be reported in relation to evaluation aims.

*Process evaluations should summarise or refer to the main findings of the trial.* To aid interpretation of evaluation findings, trial and evaluation reports should cross-reference each other and process evaluations should summarise the main trial findings.

Table 2 maps our proposed design and reporting framework to three process evaluations that were broad and detailed in reporting their process evaluation and that we judged to significantly add understanding to the main trial findings. These vary considerably in their purpose, whether they were prospective or retrospective, the processes examined and the methods used. We believe our framework helps clarify what kind of process evaluation they were, including the processes they do not examine (which is not always explicit in the original papers).

**Table 2:** Mapping of the reporting framework to three selected process evaluations

	**Summary of trial being evaluated**	**Clearly labelled as a process evaluation**	**Stated purpose**	**Processes examined**	**Specify timing**	**Methods used**	**Choice of method justified**	**Report main findings of trial**
Nazareth and colleagues [30]	Cluster-randomised trial of a pharmacist-led educational outreach intervention to improve GPs’ prescribing quality	Yes	’To describe the steps leading to the final primary outcome and explore the effect of the intervention on each step of the hypothesised pathway of change in professionals’ prescribing behaviour’	Cluster recruitment, Delivery to clusters, Adoption and Delivery to target population, Quantitative associations with effectiveness (reported in main trial paper)	Retrospective/ *post hoc*	Reporting of proportion of practices recruited. Association between proportion of GPs in each practice attending education outreach, the intervention and change in prescribing. Mixed-methods assessment of barriers and facilitators to adoption and delivery to target population.	Partly	Trial design, intervention, targeted outcomes and results summarised, main trial paper referenced
Byng and colleagues [11]	Cluster-randomised trial of a complex intervention to promote shared care for people with severe mental health problems	Yes	’To unpick the complexity of the intervention by examining interactions between components and context and then further defining its core functions’	Adoption, Reach and Delivery to target population, Qualitative associations with effectiveness	Retrospective data collection, unclear if planned prospectively	Realistic evaluation, qualitative case study, analysis of interview data with a purposive sample of participating mental health team-practice cases. Case study findings were used to better understand how the intervention changed practice and targeted outcomes.	Yes	Trial design, intervention and results summarised, main trial paper referenced
Fretheim and colleagues [33]	Cluster-randomised trial of a multifaceted intervention (educational outreach, audit and feedback, computerised reminders, patient information) to improve GPs’ prescribing quality	Yes	’The main objective of this analysis was to identify factors that could explain variation in outcomes across practices’	Delivery to clusters, Adoption and Quantitative associations with effectiveness	Prospective / pre-specified	Quantification of GP perceptions of the intervention and the trial, and pharmacist assessment of the quality of educational outreach. Regression analysis of associations with change in prescribing.	Partly	Trial design, intervention, targeted outcomes and results summarised, main trial paper referenced

GP, general practitioner.

## Conclusion

There are a number of approaches to the design of process evaluations of randomised trials in the literature [1-3,11,19,23,26,27,31,35], [37] and other additional relevant literature related to public health interventions where randomisation is often not used. Our framework pulls many smaller design considerations together into a comprehensive overview of candidate elements that are potentially relevant to the design of process evaluations of cluster-randomised trials of complex interventions. The framework draws attention to a wider range of processes that can be evaluated than many of the existing approaches, which only apply to some of these elements, although they do offer useful ways of examining these in depth. For example, normalisation process theory [35] maps well to some candidates of our framework, such as ‘response of clusters’, ‘delivery to individuals’ and ‘maintenance’, and facilitates a more in-depth sociological exploration of these candidates. Hardeman and colleagues’ causal modelling approach [37] maps well to ‘response of individuals’ and facilitates a more in-depth psychological exploration of this candidate. The RE-AIM framework focuses on the activity of clusters, but pays less attention to how clusters themselves respond to an intervention directed at them, to how patients respond to changes in care, or to unintended consequences [19]. We believe it is useful for designers of cluster-randomised trials of complex interventions to start with a broad overview of all potential candidates, and to clearly justify why some candidates are selected for detailed examination, and the methods and theories chosen to examine selected processes.

Further work is required to develop critical appraisal instruments for process evaluation studies; our proposed criteria for reporting process evaluations represent minimal requirements. We believe they will be helpful whilst more empirically-based criteria for quality assessments are developed, as have been established for the reporting [28] and quality assessment of trials [42].

There is no single best way to conduct a process evaluation, and researchers will usually need to make choices about which research questions to focus on and which methods to use. We recommend that researchers explicitly state the purpose of their process evaluation and demarcate its limits by clearly stating which processes are being examined. We propose our framework to facilitate this. Although we focus on process evaluations of cluster-randomised trials, elements of the framework are applicable to other study designs for evaluating complex interventions. Process evaluations are complex, diverse and face differing constraints based on the main trial context and study funding. However, a more structured approach to process evaluation design and reporting will improve the validity and dissemination of such studies.

## Abbreviations

CONSORT: Consolidated Standards of Reporting Trials;DQIP: Data-driven Quality Improvement in Primary Care;RE-AIM: Reach Efficacy Adoption, Implementation and Maintenance

## Competing interests

The authors declare that they received no support from any organisation for the submitted work. RF has received funding for unrelated research with Reckitt Benckiser Group Plc in the previous 3 years. The authors declare that they have no competing interests.

## Authors’ contributions

AG and BG were involved in the initial conceptualisation and design. AG reviewed the literature, carried out the analysis and interpretation of the data and contributed to the design of the model. AG prepared the first manuscript and is responsible for this article. BG, ST and RF contributed to the analysis, interpretation of data, and design of the model. BG, TD and AG designed the final framework model. All authors iteratively commented on successive drafts of the manuscript. All authors read and approved the final manuscript.
